# Surface-Modified Bilosomes Nanogel Bearing a Natural Plant Alkaloid for Safe Management of Rheumatoid Arthritis Inflammation

**DOI:** 10.3390/pharmaceutics14030563

**Published:** 2022-03-03

**Authors:** Mohammed H. Elkomy, Nabil K. Alruwaili, Mohammed Elmowafy, Khaled Shalaby, Ameeduzzafar Zafar, Naveed Ahmad, Izzeddin Alsalahat, Mohammed M. Ghoneim, Essam M. Eissa, Hussein M. Eid

**Affiliations:** 1 Department of Pharmaceutics, College of Pharmacy, Jouf University, Sakaka 72341, Saudi Arabia; nkalruwaili@ju.edu.sa (N.K.A.); melmowafy@ju.edu.sa (M.E.); khshalabi@ju.edu.sa (K.S.); azafar@ju.edu.sa (A.Z.); nakahmad@ju.edu.sa (N.A.); 2 UK Dementia Research Institute Cardiff, School of Medicine, Cardiff University, Cardiff CF24 1TP, UK; alsalahati@cardiff.ac.uk; 3 Department of Pharmaceutical Chemistry and Pharmacognosy, Faculty of Pharmacy, Applied Science Private University, Amman 11931, Jordan; 4 Department of Pharmacy Practice, Faculty of Pharmacy, AlMaarefa University, Ad Diriyah 13713, Saudi Arabia; mghoneim@mcst.edu.sa; 5 Department of Pharmaceutics and Industrial Pharmacy, Faculty of Pharmacy, Beni-Suef University, Beni-Suef 62511, Egypt; essam.mohamed@pharm.bsu.edu.eg (E.M.E.); hussien.eid@pharm.bsu.edu.eg (H.M.E.)

**Keywords:** berberine, inflammation, bilosomes, chitosan, rheumatoid arthritis, nanogel, carrageenan-induced paw edema

## Abstract

Rheumatoid arthritis (RA) is a chronic inflammatory illness affecting the joints. The characteristic of RA is gradual joint deterioration. Current RA treatment alleviates signs such as inflammation and pain and substantially slows the progression of the disease. In this study, we aimed to boost the transdermal delivery of berberine (a natural product) by encapsulating it in chitosan, surface-modified bilosomes nanogel for better management of the inflammation of RA. The chitosan-coated bilosomes loaded with berberine (BER-CTS-BLS) were formulated according to the thin-film hydration approach and optimized for various causal variables, considering the effect of lipid, sodium deoxycholate, and chitosan concentrations on the size of the particles, entrapment, and the surface charge. The optimized BER-CTS-BLS has 202.3 nm mean diameter, 83.8% entrapment, and 30.8 mV surface charge. The optimized BER-CTS-BLS exhibited a delayed-release profile in vitro and increased skin permeability ex vivo. Additionally, histological examination revealed that the formulated BLS had no irritating effects on the skin. Furthermore, the optimized BER-CTS-BLS ability to reduce inflammation was evaluated in rats with carrageenan-induced paw edema. Our results demonstrate that the group treated with topical BER-CTS-BLS gel exhibited a dramatic reduction in rat paw edema swelling percentage to reach 24.4% after 12 h, which was substantially lower than other groups. Collectively, chitosan-coated bilosomes containing berberine have emerged as a promising therapeutic approach to control RA inflammation.

## 1. Introduction

Rheumatoid arthritis (RA) is a chronic inflammatory disease that affects about 0.5–1% of the global population and is marked by dysfunction in the immune system [1]. As the disease develops, signs such as edema, inflammation, and pain may occur. Smoking, gender, age, obesity and hereditary characteristics are all contributors to the development of RA, but the hallmark of RA is increasing joint destruction [2]. Unfortunately, there is currently no cure for RA. Nevertheless, existing RA therapy alleviates symptoms, such as inflammation and discomfort, and slows down disease progression [3]. While current therapies for RA have demonstrated considerable effectiveness, 30% of the individuals who receive medication remain unresponsive to the first-line therapy. For instance, during a one-year treatment period, 30% of individuals acquire methotrexate intolerance due to serious side effects and systemic problems [4]. The development of novel natural medicines with enhanced therapeutic effectiveness and potential to provide aggregated and selective targeting of afflicted tissues is greatly on demand.

Metabolism-mediating drugs have evolved into a viable therapeutic strategy for RA owing to the high proliferative needs of RA-inflamed synovium [5]. Several metabolism-mediating drugs have been effectively used to reduce disease activity [6]. For instance, berberine, a natural alkaloid from the *Coptis Chinensis* herb, has various pharmacological properties [7]. Numerous experimental investigations have been conducted to investigate its metabolism-regulating function [8], as well as its anti-rheumatoid impact [9,10,11]. Berberine can be a powerful tool in RA therapy due to its efficacy in slowing RA progression by targeting mitochondrial oxidative phosphorylation [10].

Transdermal delivery systems (TDS) have been proposed as a potential alternative route for medication administration due to their many advantages over oral and injectable delivery systems [12]. Generally, considerable focus has been put on lipid-based vesicles as enhancers of drug penetration through the skin [13]. The vesicular system has been shown to have an excellent potential for attenuating the stratum corneum (SC) intercellular lipid barrier of the skin [14], allowing for drug diffusion through skin layers. Bilosomes (BLS) are lipid nanostructures, comprised mostly of amphiphilic bile salts, with a unique capacity to penetrate biological membranes such as those in the intestine and skin [15]. The inclusion of bile salts impedes nanocarrier degradation in the gastrointestinal tract, leading to enhanced permeation, which is beneficial for oral delivery [16]. In addition, transdermal delivery is boosted, owing to the high flexibility of BLS, which enhances the penetration through the SC and skin-deep layers [16]. Moreover, compared to traditional liposomes, the inclusion of certain bile salts, such as sodium deoxycholate (SDC), boosts the colloidal stability of the system [17]. It also has a nanosized diameter and a fluidizing effect, which enhances transdermal delivery [18,19]. Several studies have used BLS to improve the transdermal delivery of drugs such as lornoxicam [18], tizanidine hydrochloride [20], and ondansetron hydrochloride [21]. 

Chitosan (CTS), produced via the partial deacetylation of chitin, is a cationic amino polysaccharide obtained from squid pens, crustacean exoskeletons, and fungi [22]. CTS recently received much attention due to its unique attributes, such as its polycationic character, biocompatibility, in vivo biodegradability, mucoadhesiveness, and solubility at a low pH in an aqueous medium, wound healing, and antibacterial activity [23,24]. Further, CTS has been employed as a coating agent for various nanovesicles. The coating process is often facilitated by electrostatic interaction between the negative surface charge of the nanovesicles and the amino-acid-positive group of CTS [25]. The surface coating of nanovesicles by CTS has various benefits, such as boosting stability, prolonging drug release, increasing cellular absorption, and preventing drug leakage [22]. In the TDS area, CTS is attracting considerable interest, and several studies have suggested utilizing CTS as a strategy to improve TDS [26,27]. CTS has the potential to enhance mucosal barrier permeability (including ocular [28], intestinal [29] and nasal mucosae [30]) in addition to the skin barrier permeability [31]. CTS may interact with the negative load in the SC owing to its positive charge; it is therefore considered a promising candidate for the reliable delivery of BER to the deep layers of the skin.

Hydrogels are three-dimensional networks of hydrophilic polymers that can absorb water or biological fluids in vast amounts [32]. They are interesting vehicles for drug delivery applications because of their unique physical features, including biocompatibility, high porosity, biodegradability, flexibility, and controlled drug release [33]. Numerous hydrogel platforms can be employed in functional tissue models, including cellular encapsulation, hydrogel sandwich systems, fibrous hydrogel scaffolds, porous hydrogels, microfluidics, bio-printed scaffolds, hydrogel microparticles and microwells [33]. Because of their porosity, drugs may be trapped in the hydrogels and released at a frequency that depends on the molecule diffusion coefficient through the hydrogel network [34]. Furthermore, they do not affect the metabolic processes and medications may readily flow through the hydrogels [35].

Until now, no study has been conducted to assess the efficacy of topically applied, surface-modified bilosomes nanogels containing berberine in alleviating RA inflammation. As a result, three main goals were the focus of this research. First, to formulate BER-CTS-BLS using a Box–Behnken design and to choose an optimal formulation with a high desirability index. Second, to perform ex vivo and in vitro testing on the optimized formulation to assess its safety and appropriateness for transdermal administration. Finally, to test the anti-inflammatory efficacy of the optimized chitosan-coated bilosomes formulation in vivo in carrageenan-induced paw edema.

## 2. Materials and Methods

### 2.1. Materials

Berberine chloride (BER), Cholesterol, Soybean Lecithin (SL), Sodium deoxycholate (SDC), Chitosan (CTS, MW: 150,000) and dialysis bags (MW cut off: 12,000 Da), Chloroform (HPLC), and Methanol (HPLC), Carrageenan, Acetonitrile (HPLC), and Carbopol 974 NF were procured from Sigma-Aldrich (St. Louis, MO, USA). The other chemical substances and solvents used were of analytical quality.

### 2.2. Methods

#### 2.2.1. Design and Optimization of Experiments

Three causal factors at three discrete levels were selected and optimized using Box–Behnken (BB) design to reduce the size of BER-CTS-BLS vesicles while increasing surface charge and entrapment. Design assessment and selection of the optimized formulation were carried out via Design Expert^®^ software (Version 12.0.3.0, Stat-Ease Inc. Minneapolis, MN, USA). Seventeen formulations were compounded, including twelve concerning the midpoint of each edge of a tri-dimensional cube, and five concerning the center point replication. Three causal factors were processed: lipid level (%*w*/*v* with respect to total dispersion) (X_1_), SDC level in lipid mixture (%*w*/*w*) (X_2_), and CTS level (%*w*/*v*) (X_3_). Particle size (Y_1_: PS), Entrapment efficiency (Y_2_: EE %), and Zeta potential (Y_3_: ZP) were selected as the response variables. Table 1 and Table 2 detail the levels of independent variables and the composition of the different experimental runs created using the BB design.

#### 2.2.2. Preparation of Berberine-Loaded Chitosan-Coated Bilosomes (BER-CTS-BLS)

BER-CTS-BLS were formulated using the thin-film hydration technique [36]. In brief, SL, SDC, cholesterol (20 mg) and BER (10 mg) were dissolved in a round-bottom flask in chloroform mixed with methyl alcohol (2:1). The organic solution obtained was evaporated through a rotary evaporator (Stuart rotary evaporator, RE300, North Yorkshire, UK) under reduced pressure at 40 °C until a thin dry film was produced. The produced lipid film was exposed to hydration by adding phosphate buffer saline (PBS, 10 mL, pH 7.4) to the flask that was allowed to rotate under normal pressure at 100 rpm for 1 h. A bath sonicator (Sonix TV ss-series ultrasonicator, North Charleston, SC, USA) was used to sonicate the dispersion for 20 min. For BER-CTS-BLS preparation, a specific amount of CTS was dissolved in 0.5% acetic acid solution (% *v*/*v*). Then, 2 mL CTS solution was added to the above dispersion dropwise under stirring (0.2 mL/min, 100 rpm, and 25 °C) [37]. Finally, the formulation was stored in a refrigerator (4 °C) until further assessment.

#### 2.2.3. Chromatographic Conditions

The quantity of BER was estimated using an adopted HPLC method [38]. Agilent Eclipse C_18_ column (4.60 mm × 25 cm, i.d., 5 μm PS) was used to detect BER quantity. A mobile phase of 0.05 mol/L NaH2PO4 and acetonitrile (70:30 *v*/*v*) (pH adjusted with phosphoric acid to 2.5) was pumped at a 1 mL/min flow rate through the HPLC system. UV detection was carried out at 345 nm at 30 °C. The injection volume and retention time were 20 µL and 5 min, respectively. The employed HPLC method for BER quantitation was highly sensitive over 0.01–1 µg/mL (R^2^ = 0.999).

#### 2.2.4. Characterization of the Experimental Runs

##### Particle Size and Surface Charge Analysis

A Zetasizer 2000 (Malvern Instruments Ltd., Malvern, UK) was utilized to estimate BER-CTS-BLS particle diameter and surface charge using dynamic light scattering [39]. The formulated bilosomal vesicles were diluted with PBS before being used in the study. Three sets of measurements were made, and the mean results were reported.

##### BER Entrapment

The amount of BER held within the formulated preparation was estimated indirectly by subtracting the amount of non-entrapped BER (free BER) from the amount of BER initially added (10 mg). The dispersion was centrifuged at 14,000 rpm (4 °C, 3 h) in a cooling centrifuge (SIGMA 3-30K, Steinheim, Germany) to separate the supernatant containing free BER [40]. The supernatant was diluted and subjected to the HPLC system described above to determine BER concentration. The following equation determined the drug’s EE percentage:

(1) $$\mathrm{EE}\;\%=\frac{\mathrm{Total}\;\mathrm{amount}\;\mathrm{of}\;\mathrm{BER}-\mathrm{free}\;\mathrm{BER}}{\mathrm{Total}\;\mathrm{amount}\;\mathrm{of}\;\mathrm{BER}}\times 100\;$$

#### 2.2.5. Optimized BER-CTS-BLS Characterization

##### Ex Vivo Skin Permeation Study

Male Wistar rats (200–210 gm) were euthanized, the hair on the abdomen skin was gently clipped away using electric clippers, and then the dorsal skin was excised. Subcutaneous tissues and adherent fats were carefully scraped away until skin thickness was approximately 1 to 1.5 mm. The skin was then sliced into appropriately sized pieces and immersed in 0.9% normal saline for 1 h. The dissected rat abdomen skin was put between the donor and receptor compartments on Franz diffusion cells (5 cm^2^), with the SC side looking upward and the dermal side facing downward [40]. Two milliliters of the optimized BER-CTS-BLS and BER solution (each containing 2 mg) was added to the donor chamber. Under magnetic stirring (50 rpm, 37 °C), fifty milliliters of PBS (pH 7.4) were put into the receptor chamber. At preset intervals, one milliliter of PBS was collected from the receptor compartment and substituted with one milliliter of fresh PBS. The collected samples were filtered and analyzed with the HPLC system described above. Permeation parameters were estimated according to a previously published method [40].

##### In Vitro Release Evaluation

In vitro release experiments were conducted using Franz diffusion cells (5 cm^2^ surface area). A cellulose dialysis membrane was used to separate the donor chamber from the receptor chamber [41]. The donor chamber contained a particular volume of the optimized BER-CTS-BLS and BER solution (equivalent to 3 mg). Fifty milliliters of PBS were introduced to the receptor chamber with constant stirring (50 rpm, 37 °C) [42]. At preset intervals, one milliliter from the receptor compartment was taken and replaced with one milliliter of fresh PBS. The collected samples were filtered and analyzed with the HPLC system described above. 

##### Morphological Evaluation

The morphological investigation was conducted on the optimized BER-CTS-BLS formulation. One drop of freshly formulated, diluted BER-CTS-BLS was placed on a carbon-coated grid and then left for 5 min at 25 °C until dryness. Phosphotungstic acid (0.1 mL, 1% *w*/*v*) was added to the grid, left for 2 min to permit sufficient stain absorption, and then a filter paper was added to withdraw excess liquid. The samples were examined using TEM (Jeol, Tokyo, Japan) that operates at a constant voltage of 80 kV [43].

##### Stability Study of the Optimized BER-CTS-BLS

Stability testing was performed on the optimized BER-CTS-BLS formulation by keeping it for three months in a glass vial (4 °C). Samples from the optimized BER-CTS-BLS were collected after formulation and then after specified time intervals for three months. Particle diameter and entrapment of the collected samples were determined [40].

#### 2.2.6. Formulation of BER-CTS-BLS-Based Gel

The optimized BER-CTS-BLS formulation and free BER solution were mixed with Carbopol 974 NF polymer to make a gel, according to a previously reported method [40]. Carbopol 974 NF (2% *w*/*w*) and preservatives (0.01% propylparaben and 0.1% methylparaben) were sprinkled under stirring in water for 2 h. BER-CTS-BLS were then added to the gel base (1% *w*/*w* of BER), followed by 1 h stirring. Using Triethanolamine, the pH was changed to 6.0 ± 0.05 to achieve BER-CTS-BLS gel with sufficient strength for topical use. The homogeneity, spreadability, clarity and rheological characteristics of the produced gels were all evaluated. The characterization of the produced gels was discussed previously [44].

#### 2.2.7. Animal Experiment

In this research, male Wistar rats (220–250 g) were utilized. The rats were kept in a controlled environment (temperature, relative humidity, and lighting). All the experiments were performed following the approval of the Local Institutional Animal Ethics Committee at Beni-Suef University (Acceptance No: 022-226) and were conducted according to the Guide for the Care and Use of Laboratory Animals published in 2011 by the United States National Academy of Sciences.

##### Histopathological Investigation of the BER-CTS-BLS-Based Gel

The histopathological investigation was conducted to assess the safety of the topical application of the BER-CTS-BLS gel. Six rats were randomly assigned to two groups. Group A acted as a control, while the other group received the optimized BER-CTS-BLS gel topically on the hairless dorsal skin surface for seven days. Following that, the rats were killed, and the skin was removed for histological examination. The detached skin was fixed in formaldehyde (10%) for 24 h. The sections were cleaned in xylene and then put in blocks of paraffin wax for 24 h at 56 °C. The sections were sliced into five µm thick slices, then placed on glass slides and stained with hematoxylin and eosin (H&E) [45]. Finally, a light microscope was used to inspect the stained slices.

##### Anti-Inflammatory Effectiveness

The anti-inflammatory effects of the various formulations were investigated using a carrageenan-induced paw edema model [46]. Twenty-five rats were split into five equal groups. The first group acted as a control (untreated). The second group received Voltaren^®^ emulgel treatment (as a reference standard). The third group received topical CTS-BLS gel treatment (no drug). The fourth group received free BER topical gel (1% BER). The fifth group received topical BER-CTS-BLS gel (1% BER). A total of 0.5 gm of each formulation was gently rubbed with the index finger (50 times). Thirty minutes before the treatments, paw edema was induced by injecting the animal groups with 0.1 mL carrageenan solution (1% *w*/*v*) into the intraplanar area of the right paw [40]. The paw volume was measured using a digital caliber before and after different treatments at specified time intervals. The following equation was used to obtain the percent of swelling:

where V_t_ denotes the hind paw volume at time t after treatment application, and V_0_ is the hind paw volume at time 0 (before treatment application).

(2) $$\mathrm{Swelling}\;\left(\%\right)=\left(\frac{\mathrm{Vt}-V_{0}}{V_{0}}\right)\times 100$$

#### 2.2.8. Statistical Analysis

Each experiment was performed three times. Statistical differences between groups were evaluated where appropriate using one-way ANOVA with Tukey post hoc test, as incorporated in the aov function in R software (version 3.6.2, R Core Team, 2019). Throughout the research, a difference was considered significant if the *p*-value was less than 0.05.

## 3. Results and Discussion

### 3.1. Bilosomes Formulation

Initial experiments were conducted to determine the effect of various factors including sonication duration, hydration medium, lipid content, and drug concentration on BLS formation. All variables have been developed to obtain a suitable BLS size with the highest entrapment. In reality, BLS vesicles were prepared using SL combined with cholesterol to boost vesicle entrapment and stability [47,48,49]. SDC was used in various concentrations as an edge activator. Collectively, these circumstances were optimal for vesicle preparation: PBS (pH 7.4) as the hydration medium, chloroform-methanol mixture as the organic solvent, 20 mg of cholesterol, 10 mg of BER, 20 min sonication, and 1 h hydration.

### 3.2. Experimental Design and Statistical Analysis

**Figure 1:**
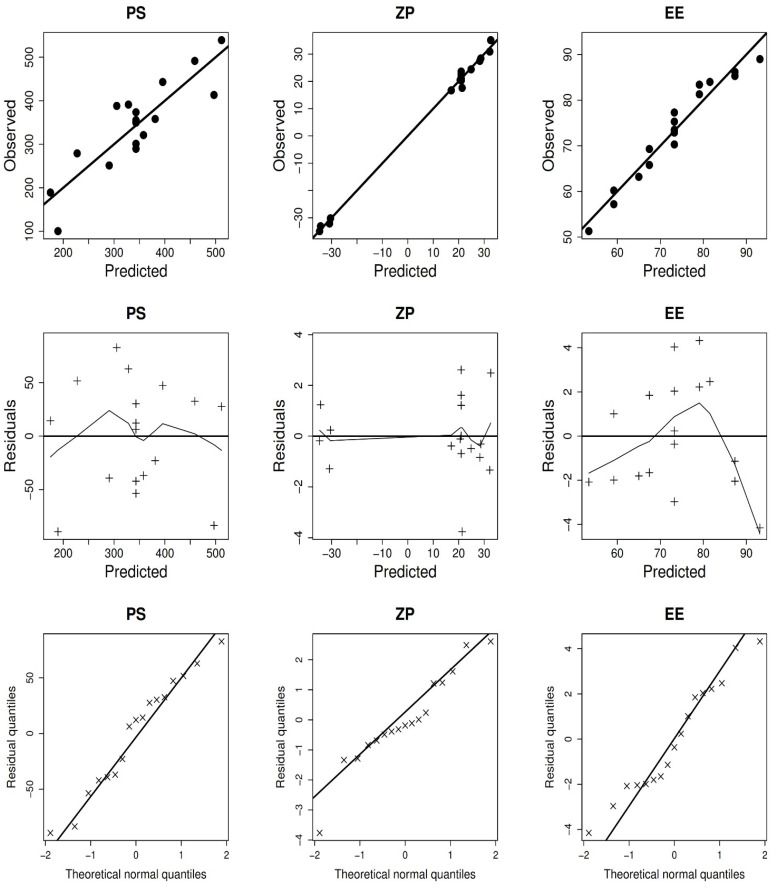
The diagnostic graphs of the response variables models.

**Figure 2:**
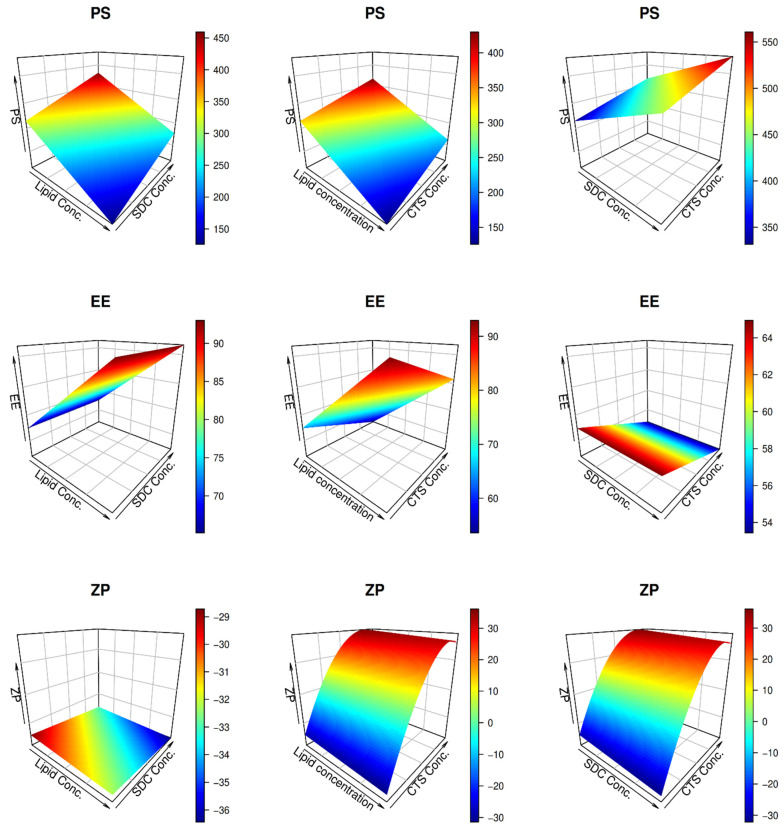
RSM plots for the impacts of the independent variables on the response variables concerning the optimization of BER-CTS-BLS.

As confirmed by the non-significant lack of fit error, the variability of the observed data was well characterized by the models (Table 3). Visual examination of the model diagnostic graphs (Figure 1) shows a good fit of the data, with no discernible patterns of residual errors that mostly followed a normal distribution. Figure 2 depicts the estimated connection between independent and dependent factors, with each panel displaying the interaction of two independent variables, with the third set at the center value.

#### 3.2.1. Effect of Independent Variables on PS

As reported in Table 2, the size of the formulated nanovesicles was between 100.3 and 539.1 nm. A linear model was shown to be acceptable for the presented PS data using ANOVA, and the impact of lipid, SDC, and CTS on the size of BER-CTS-BLS was very significant (*p <* 0.05).

The PS strongly affects the permeation of vesicles across the skin, and thus, during BER-CTS-BLS formulation, small PS is very desirable. Surprisingly, increasing the lipid content reduced the PS of BER-CTS-BLS (Figure 2, upper panel). These findings may be attributed to increased surface area at high lipid levels, allowing more BER to be housed in the bilayer vesicle [50,51]. The high quantity of drug captured in the vesicles shows improved vesicular membrane packaging and consequent reduction in size [52].

The effect of SDC levels on PS was investigated, and it was observed that the size of BER-CTS-BLS increased as the amount of SDC increased.

The anionic nature of SDC, which results in substantial steric repulsion between bilayers, may explain these results [53,54]. Furthermore, the SDC steroid-like structure may boost the bulkiness of the BLS, thus increasing the particle size [55]. The aforementioned results contrast previous studies that indicated the increasing SDC concentration resulted in a reduction in PS. This was attributed to the impact of SDC on vesicle membrane curvature [56,57].

The size of BER-CTS-BLS increases as the CTS content increases, similar to the SDC effect. The coating layer produced on the surface by CTS is the main cause of PS growth [26,58].

#### 3.2.2. Effect of Independent Variables on EE

As illustrated in Table 2, BER entrapment was in the range of 51.3–89% for all vesicles. A linear model was shown to be acceptable for the presented entrapment efficiency data using ANOVA. In the middle panel of Figure 2, the effect of lipid, SDC, and CTS levels on entrapment is shown graphically. Factorial variance analysis showed that the concentration of lipids and CTS positively impacted entrapment (*p <* 0.05). It is easy to distinguish that the higher the lipid level, the higher the entrapment of BER by reviewing the middle panel of Figure 2 [50,59].

Increasing the CTS level from 0 to 0.25%, conversely, significantly reduced the entrapment (Figure 2, middle-right panel). This negative impact is explained by the positive charges on both BER and CTS, which resulted in electrostatic repulsion and fierce competition for phospholipid affinity. Consequently, the entrapment of BER was reduced [27,58].

Although the bile salt had an insignificant effect on entrapment (Figure 2, middle panel), some reports have shown that the entrapment of BLS declined as the concentration of SDC increased. The fluidizing impact of SDC on the bilayer membrane of vesicles, which induces drug leakage, may be responsible for these findings [60]. Additionally, at high levels, the bile salt may form mixed micelles, thus increasing the solubility of the medication in the dispersion medium and lowering the entrapment [61,62].

#### 3.2.3. Effect of Independent Variables on ZP

As illustrated in Table 2, the ZP of formulated nanovesicles ranged between (−) 34.9 and (+) 35.1 mV. ANOVA analysis of the provided ZP data demonstrates the sufficiency of a quadratic model. Furthermore, ANOVA analysis of the obtained response variables shows that the impact of lipid, SDC, and CTS on BER-CTS-BLS surface charge was very significant (*p <* 0.05). 

The lower panel in Figure 2 indicates that the higher the lipid concentration, the higher the ZP negative charge. The negative phosphate groups (PO_3_^−^) of lecithin are responsible for this increase in the negative charge [63]. Furthermore, owing to the negative charge of the edge activator (SDC), a high SDC level is accompanied by an increase in the ZP negative charge of the formed vesicles [64]. Finally, due to the positive charge of CTS, the presence of CTS in the BLS modified the ZP values from negative to positive [63].

#### 3.2.4. Formulation Optimization

After applying constraints on the response variables, the Design^®^ Expert program recommended formulating an optimal BER-CTS-BLS formulation with total desirability of 0.88. The optimum formulation variables were expected to be 5% lipid concentration, 5% SDC concentration, and 0.16% CTS concentration. The optimum response factors were PS of 188.5 nm, entrapment of 86.9%, and ZP of 28.3 mV (Table 4). Our models successfully predicted the features of the optimum BLS (prediction error < 9%), as shown in Table 4.

### 3.3. Optimized BER-CTS-BLS Characterization

#### 3.3.1. Ex Vivo Skin Permeation Study

**Figure 3:**
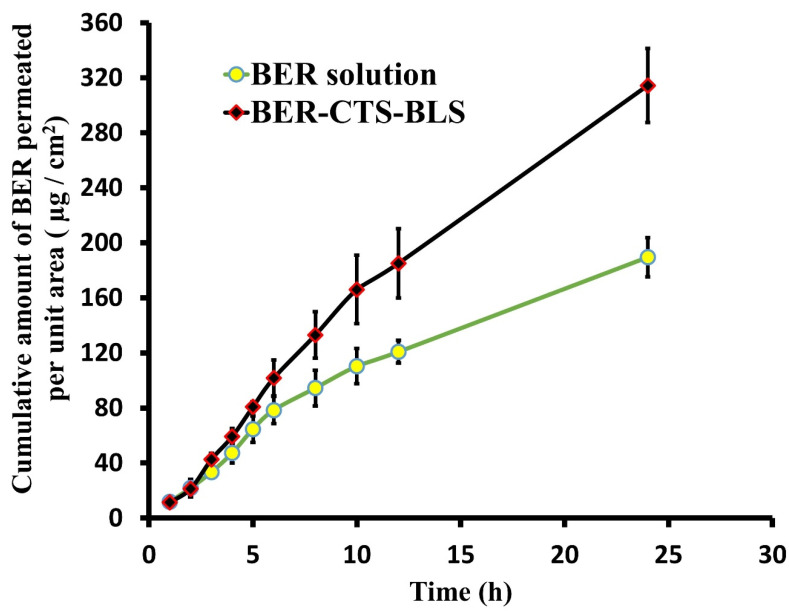
Ex vivo permeation of berberine from BER-CTS-BLS (optimal formula) and BER solution through rat skin.

Figure 3 illustrates BER permeation profiles from BER-CTS-BLS and BER solution. The permeation profiles of the BER-CTS-BLS and BER solution varied significantly (*p* < 0.05). As shown in Table 5, BER-CTS-BLS permeation coefficient was approximately 1.5-fold that of BER solution, indicating improved BER penetration when loaded in CTS-coated BLS. Numerous factors can be counted in the increased penetration of BER-CTS-BLS. Firstly, it was defined that phospholipids have an affinity for biological membranes [65,66], and they increase hydration when they come into contact with SC [49]. As a result, the packed lipid structure of the skin loosens up temporarily, enabling the drug to pass through more readily [59]. Second, the surface charge of BER-CTS-BLS formulation is crucial in elucidating how the CTS might improve transdermal transport [31,67]. CTS coating supplied the positive charge on the BLS surface, which was critical in interacting with the negative charge of SC to facilitate drug diffusion. Third, the potential of CTS positive charge to disrupt the negative charge tight junctions in the skin to enable BER-CTS-BLS distribution [68]. Finally, the CTS’s bio-adhesion force enhances the vesicle’s contact duration with the skin, resulting in higher diffusion and, therefore, higher penetration [27].

#### 3.3.2. In Vitro Release Evaluation

**Figure 4:**
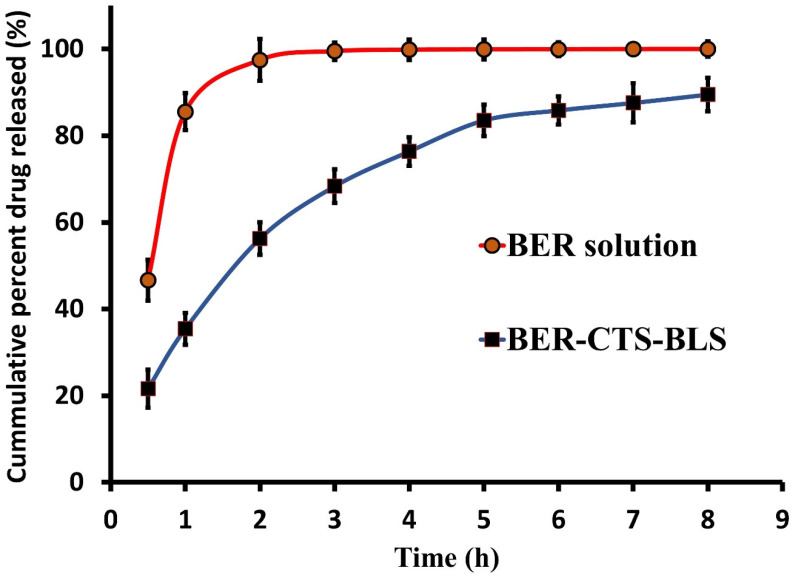
In vitro release profile of berberine from BER-CTS-BLS (optimal formula) and BER solution.

The release profile of BER-CTS-BLS and BER solution varied significantly (Figure 4, *p >* 0.05). Within 3 h, the BER solution released 99.43% of the accumulated amount, compared to 68.32% for the BER-CTS-BLS solution. Accordingly, the rate of BER released from BER-CTS-BLS was slower than the rate of BER released from the corresponding BER solution, indicating that the bilosomal system had the capacity to delay the release of BER. Moreover, BER released from the BER-CTS-BLS exhibited an early-burst phase lasting 3 h, followed by a sustained phase lasting 8 h and reaching 89.49% of BER. These results are consistent with previously published studies [69].

#### 3.3.3. Morphological Evaluation

**Figure 5:**
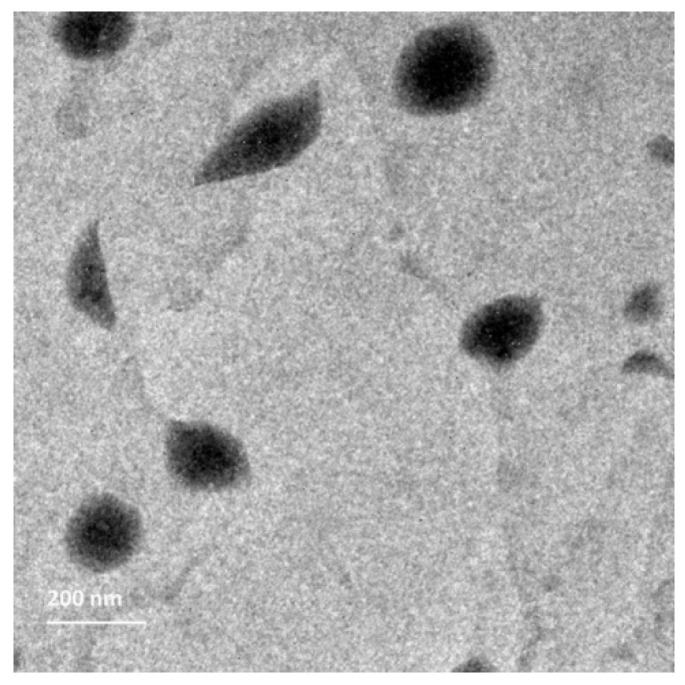
Transmission electron microscopy micrograph of the optimized BER-CTS-BLS formulation.

TEM investigation was used to explore the morphological characteristics and PS of the optimized BER-CTS-BLS dispersion. Figure 5 depicts homogenous unilamellar vesicles that are not aggregated and are almost spherical in shape. Furthermore, the surface of the bilosomes had a thin layer of CTS coating.

#### 3.3.4. Stability Study of the Optimized BER-CTS-BLS

**Figure 6:**
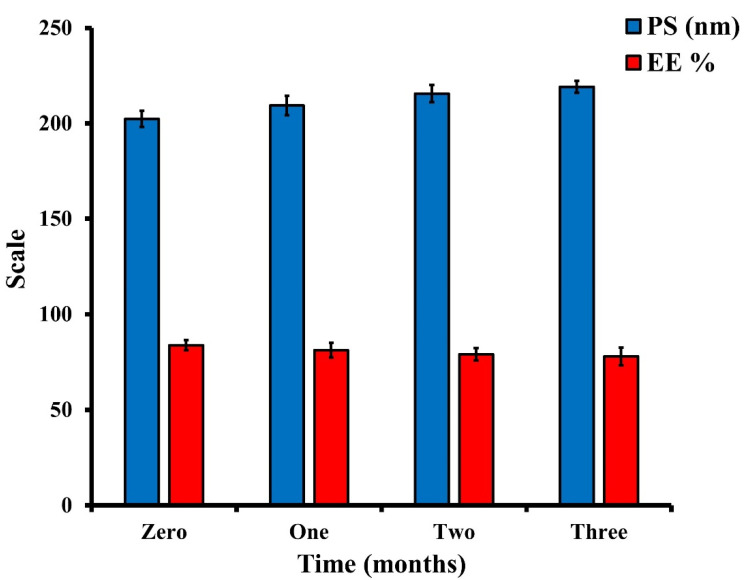
Particle size and entrapment efficiency analysis of the optimized BER-CTS-BLS preparation during three months of storage (4 °C).

After 30, 60 and 90 days, BER entrapment and particle size of the optimal formulation were determined. The BER-CTS-BLS formulation stability was excellent, with no layer separation or sedimentation. Indeed, a little reduction in entrapment efficiency and a slight increase in vesicle diameter were detected but were determined to be negligible (*p >* 0.05), suggesting that the vesicle diameter remained stable throughout the storage time (Figure 6). A dispersion is stable if its surface charge value is high (negative or positive), thus preventing flocculation development and particle aggregation. Stable formulations often have surface charge higher than or equal to +30 mV or less than or equal to −30 mV [70,71]. The optimized BER-CTS-BLS formulation has a surface charge of 30.8 ± 2.4 mV, suggesting dispersion stability. Moreover, the bilosomal membrane is stabilized by the existence of cholesterol [49].

### 3.4. Histopathological Study

**Figure 7:**
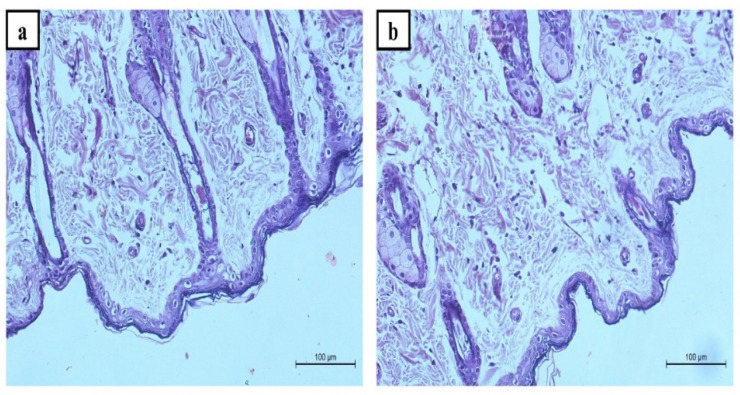
Representative histopathological images of untreated rat skin group (**a**) and BER-CTS-BLS gel treated rat skin group (**b**).

Skin segments were inspected under a light microscope to ensure safety after topical application of the optimized nanoparticles. The epidermis of Group A has normal keratin-coated layers. Additionally, connective tissue and cutaneous vascularity were seen in the dermis, and skin appendages were normal (Figure 7a). When BER-CTS-BLS gel was applied to the hairless skin of rats (Figure 7b), the epidermis layer had a normal keratin squamous layer, and the dermis layer was free of erythema, edema, and inflammation. 

### 3.5. Anti-Inflammatory Effectiveness

**Figure 8:**
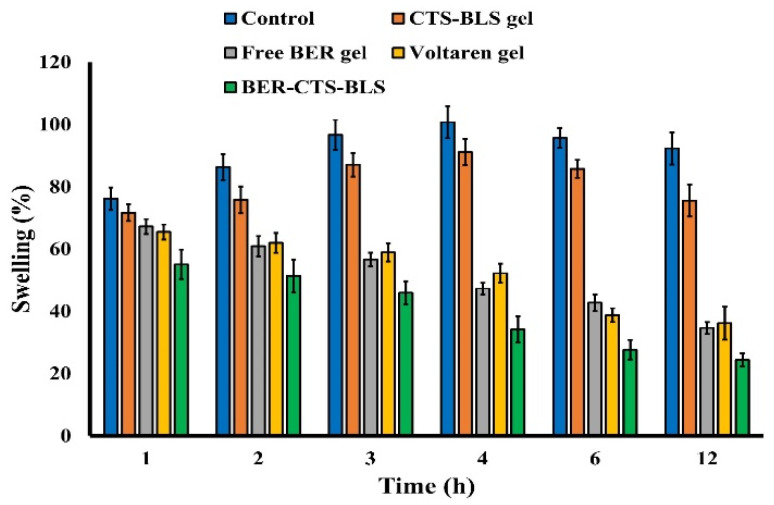
Percentage swelling in the untreated (Control), CTS-BLS gel (empty nanoparticles), free BER gel, Voltaren^®^ emulgel, and the optimized BER-CTS-BLS formulation group in carrageenan-induced paw edema rat model.

As illustrated in Figure 8, after 4 h, a significant increase in inflammation was observed in the control and CTS-BLS groups (% swelling of 100.7 and 91.1%, respectively). Furthermore, following 1 h of carrageenan injection, the group treated with Voltaren^®^ emulgel demonstrated a swelling of 65.4%, which was significantly lower than that in the control and CTS-BLS groups (*p <* 0.05). Notably, animals treated with BER formulations (free BER and BER-CTS-BLS) had a significantly lower percentage of inflammation than those treated with control or CTS-BLS (*p <* 0.05). However, no significant change was seen between the groups receiving free BER gel and those receiving Voltaren^®^ emulgel (*p >* 0.05). The BER-CTS-BLS treated group, conversely, showed a substantial reduction in the percentage of swelling after 1 h of the topical application and a swelling of 24.4% after 12 h, which is considerably lower than that observed in the other groups (*p <* 0.05). These findings demonstrate that bilosomal systems significantly enhanced BER transdermal penetration across the skin. A range of issues may explain these findings. First, the fluidizing action of the bile salts, especially SDC, label these salts as highly efficient transdermal penetration enhancers when administered via TDS [72]. Second, the CTS coating increased the contact time with the skin, therefore it enhanced drug diffusion. Third, the BER-CTS-BLS vesicle size was in nanoscale. Finally, it was shown that large concentrations of phospholipids have a strong affinity for biological membranes [65,66] and increase SC hydration [49], hence the lipid structure of the skin becomes loose temporarily, permitting the drug to penetrate more readily [59].

The capacity of BER to suppress the production of the potent inflammatory mediator PGE2 in inflamed tissue may explain the anti-inflammatory activity of BER formulations [7]. Overall, the findings show that bilosomal systems help BER penetrate the skin and provide its anti-inflammatory action.

## 4. Conclusions

This is the first trial to use chitosan-coated bilosomes loaded with berberine as a novel agent in RA inflammation therapy. The optimized nanoparticles had a size of 202.3 nm, a surface charge of 30.8 mV, 83.8% entrapment, and high stability. Ex vivo permeability and release experiment exposed that BER-CTS-BLS had greater permeability and an extended release time. Histological evaluation showed that BER-CTS-BLS gel is suitable for transdermal use. In vivo experiments revealed that the BER-CTS-BLS treated group exhibited a dramatic reduction in rat paw edema swelling percentage to reach 24.4% after 12 h, which was substantially lower than other groups. It is inferred that the BER-CTS-BLS nanogel is a promising delivery platform for transdermal administration of BER for RA inflammation control. 

## Figures and Tables

**Table 1:** The independent factor levels used for BER-CTS-BLS optimization.

Independent Factors	Levels		
	−1	0	1
X_1_: Lipid concentration (%*w*/*v*)	2.5	3.75	5
X_2_: SDC concentration in lipid mixture (%*w*/*w*)	5	10	15
X_3_: CTS concentration (%*w*/*v*)	0	0.125	0.25

**Table 2:** The composition of the different experimental runs and the results of response variables of BER-CTS-BLS formulations.

F	Lipid (% *w*/*v*)	SDC in Lipid Mixture (% *w*/*w*)	CTS (% *w*/*v*)	PS (nm)	EE (%)	ZP (mV)
1	2.50	10	0.250	413.2 ± 5.45	51.3 ± 2.6	(+) 30.9 ± 1.2
2	3.75	15	0.250	491.4 ± 3.58	65.8 ± 3.2	(+) 27.5 ± 0.6
3	3.75	10	0.125	355.3 ± 7.30	77.3 ± 4.7	(+) 20.3 ± 1.1
4	3.75	10	0.125	349.4 ± 16.12	73.5 ± 3.8	(+) 23.6 ± 0.3
5	5.00	5	0.125	189.0 ± 2.29	86.2 ± 5.3	(+) 17.6 ± 0.5
6	2.50	5	0.125	358.9 ± 6.31	60.2 ± 3.4	(+) 24.4 ± 1.7
7	3.75	5	0.000	279.8 ± 13.24	83.4 ± 5.2	(−) 30.2 ± 0.8
8	5.00	10	0.000	100.3 ± 1.06	89.0 ± 6.1	(−) 33.1 ± 2.3
9	3.75	5	0.250	391.1 ± 8.36	69.3 ± 2.2	(+) 35.1 ± 1.9
10	2.50	10	0.000	443.5 ± 11.93	63.2 ± 2.9	(−) 32.1 ± 2.7
11	3.75	15	0.000	321.0 ± 15.28	81.3 ± 3.7	(−) 34.9 ± 3.4
12	5.00	10	0.250	251.2 ± 9.87	84.0 ± 2.5	(+) 28.4 ± 1.4
13	2.50	15	0.125	539.1 ± 25.28	57.2 ± 3.1	(+) 20.5 ± 0.4
14	5.00	15	0.125	388.7 ± 12.34	85.3 ± 5.6	(+) 16.7 ± 0.3
15	3.75	10	0.125	301.0 ± 10.29	72.9 ± 3.6	(+) 21.0 ± 0.7
16	3.75	10	0.125	373.4 ± 5.92	75.3 ± 2.4	(+) 22.6 ± 1.1
17	3.75	10	0.125	289.5 ± 7.27	70.3 ± 4.8	(+) 22.2 ± 1.9

**Table 3:** Analysis of variance of the measured responses data.

Source	Size (nm)		EE%		ZP (mV)	
	F	*p*-Value	F	*p*-Value	F	*p*-Value
Model	14.45	0.0002	90.62	<0.0001	795.32	<0.0001
X_1_: Lipid concentration (%*w*/*v*)	26.43	0.0002	230.84	<0.0001	7.85	0.0160
X_2_: SDC concentration in lipid mixture (%*w*/*w*)	10.60	0.0063	1.64	0.2223	11.55	0.0053
X_3_: CTS concentration (%*w*/*v*)	6.33	0.0258	39.37	<0.0001	2511.95	<0.0001
X_3_^2^					649.94	<0.0001
Lack of Fit	3.06	0.1471	0.9911	0.5482	2.28	0.2221
Model	Linear		Linear		Quadratic	
Adjusted R^2^	0.7161		0.9438		0.9950	
R^2^	0.7693		0.9544		0.9962	
%CV	16.53		3.58		16.76	
Predicted R^2^	0.5478		0.9190		0.9920	
Adequate precision	12.2408		31.2946		69.7784	
SD	56.72		2.62		1.78	
$\mathrm{PS}=343.11-103.10.X_{1}+65.30.X_{2}+50.45.X_{3}$						
$\mathrm{EE}\;\%\;=73.26+14.08.X_{1}-1.19X_{2}-5.81.X_{3}$						
$\mathrm{ZP}=20.99-1.76.X_{1}-2.14.X_{2}+31.53.X_{3}-22.04.X_{3}^{2}$						

**Table 4:** Experimental, model expected and prediction error values of the optimized BER-CTS-BLS formulation.

Response Variables	Experimental Value	Expected Value	Prediction Error (%) *
Particle size (nm)	202.3	188.5	6.8
Entrapment (%)	83.8	86.9	3.7
Zeta potential (mV)	30.8	28.3	8.1

* Calculated as (experimental-model expected)/experimental × 100.

**Table 5:** Ex vivo permeation parameters of BER-CTS-BLS and BER solution.

Formulation	Cumulative BER Permeated at 24 h (μg/cm^2^)	Permeability Coefficient (cm/h)	Flux J_ss_ (µg·cm^−2^ h^−1^)
BER-CTS-BLS	314.5 ± 26.87	0.0037 ± 0.00025	3.69 ± 0.56
BER solution	189.5 ± 14.12	0.0026 ± 0.00032	2.71 ± 0.37
